# A prospective controlled evaluation of winterized *Pistacia lentiscus* oil nasal spray in vasomotor rhinitis

**DOI:** 10.3389/falgy.2026.1946438

**Published:** 2026-10-15

**Authors:** Alexander Bertuccioli, Annalisa Belli, Davide Sisti, Marco B. L. Rocchi, Chiara M. Palazzi, Matteo Gelardi, Ignazio La Mantia, Veronica Seccia, Simonetta Masieri, Elona Begvarfaj, Amjad Khan, Alberto Macchi

**Affiliations:** 1 Department of Biomolecular Sciences, University of Urbino “Carlo Bo”, Urbino, Italy; 2 Microbiota International Clinical Society, Turin, Italy; 3 Department of Clinical and Experimental Medicine, University of Foggia, Foggia, Italy; 4 Faculty of Medicine and Surgery, University of Enna “Kore”, Enna, Italy; 5 Department of Surgical, Medical, Molecular Pathology and Critical Care Medicine, Pisa University Hospital, Pisa, Italy; 6 Department of Oral and Maxillofacial Sciences, Sapienza University, Rome, Italy; 7 Department of Sense Organs, Sapienza University, Rome, Italy; 8 Department of Biochemistry, Liaquat University of Medical and Health Sciences (LUMHS), Jamshoro, Pakistan; 9 Ent Clinic ASST Settelaghi, University of Insubriae Varese, Varese, Italy

**Keywords:** biofilm, lentisk oil, microbial modulation, nasal cytology, non-allergic rhinopathy, PNIF, SNOT-22, vasomotor rhinitis

## Abstract

**Background:**

Vasomotor rhinitis (VMR), currently included within the broader spectrum of non-allergic rhinopathy, is a chronic condition characterized by nasal obstruction, rhinorrhea, and hyperreactivity to non-specific environmental triggers. Although traditionally considered a neurovascular disorder driven by autonomic dysregulation, emerging evidence suggests that mucosal inflammatory and microbial factors may contribute to symptom persistence in selected patients. Winterized *Pistacia lentiscus* oil has demonstrated antimicrobial and anti-biofilm properties in upper airway experimental models, suggesting potential therapeutic relevance in chronic nasal disorders.

**Methods:**

This prospective controlled study evaluated the effectiveness of a nasal spray medical device containing winterized *Pistacia lentiscus* oil (Bactorinol® nasal spray, PharmExtracta S.p.A., Italy) in patients with VMR. A total of 120 participants were enrolled, with 110 completing the study (58 treated, 52 controls). Primary endpoints included changes in Peak Nasal Inspiratory Flow (PNIF) and symptom burden assessed using the Sino-Nasal Outcome Test (SNOT-22). Secondary endpoints included nasal cytological assessment of inflammatory cellularity (neutrophils, eosinophils, mast cells), bacterial abundance, supranuclear stria, and cytological findings compatible with biofilm-like structures. Repeated-measures General Linear Models were used to evaluate treatment effects over time.

**Results:**

Compared with controls, treated patients demonstrated significantly greater clinical improvement over the study period. SNOT-22 scores decreased by 30.5% in the treatment group versus 9.7% in controls (time × group interaction, *p* < 0.001). PNIF improved by 44.5% in treated subjects compared with 20.8% in controls (*p* = 0.006 for interaction). Significant treatment-associated reductions were also observed for neutrophils (*p* = 0.001), mast cells (*p* = 0.021), bacterial abundance (*p* = 0.002), and cytological findings compatible with biofilm-like structures (*p* = 0.001). Although the treatment group presented a higher baseline burden for several parameters, post-treatment outcomes favored the intervention group.

**Conclusions:**

Winterized *Pistacia lentiscus* oil nasal spray was associated with significant improvements in symptoms, nasal airflow, inflammatory cytology, and microbial-related cytological findings in patients with VMR. These findings support the hypothesis that selected phenotypes of non-allergic nasal disease may involve mucosal inflammatory and microbial components beyond classical neurovascular dysfunction. Larger randomized studies with microbiological and molecular characterization are warranted to confirm these preliminary findings and clarify mechanisms of action.

## Introduction

Vasomotor rhinitis (VMR), more recently encompassed within the broader concept of non-allergic rhinopathy, represents one of the most prevalent forms of non-allergic rhinitis and is characterized by chronic nasal symptoms including obstruction, rhinorrhea, postnasal drip, and heightened responsiveness to non-specific environmental triggers in the absence of IgE-mediated sensitization or active infection (1–5). Despite its high prevalence and clinically significant impact on sleep quality, daytime performance, fatigue, and overall quality of life, its pathophysiology remains only partially understood (1, 2). Current evidence suggests that VMR is primarily driven by dysregulation of autonomic nasal control, with an imbalance between parasympathetic and sympathetic pathways leading to abnormal vasodilation, glandular hypersecretion, and neurogenic hyperreactivity of the nasal mucosa. Clinical assessment commonly relies on validated patient-reported outcome tools such as the Sino-Nasal Outcome Test (SNOT-22), visual analog scales, and symptom scores, which provide standardized measures of disease burden and therapeutic response (2). Objective assessment of nasal mucosal conditions may complement patient-reported outcome measures, particularly when symptoms alone do not fully reflect local mucosal alterations (6).

Beyond neurovascular dysfunction, increasing evidence suggests that chronic sinonasal inflammation may also be influenced by microbial ecosystem alterations and biofilm formation. Bacterial biofilms represent highly organized microbial communities embedded in a self-produced extracellular matrix that confers substantial resistance to host defenses and conventional antimicrobial therapies, promoting chronic mucosal inflammation and persistence of symptoms (7, 8). Although the role of biofilms has been more extensively investigated in chronic rhinosinusitis, similar mechanisms of microbial persistence and mucosal dysregulation may plausibly contribute to selected phenotypes of chronic non-allergic nasal disease, making cytological nasal assessment and biofilm-oriented monitoring clinically relevant (7, 9).

In this context, *Pistacia lentiscus* berry oil (lentisk oil), a traditional Mediterranean phytotherapeutic agent, has emerged as a potential microbial-modulating intervention. Experimental evidence suggests that lentisk oil possesses antimicrobial activity against relevant upper airway pathogens, including *Streptococcus* species and *Staphylococcus aureus*, mediated in part by its unsaturated fatty acid fraction and bioactive anacardic acid derivatives (9–14).

The winterization process allows selective fractionation of *Pistacia lentiscus* oil, potentially concentrating bioactive compounds implicated in antimicrobial and anti-biofilm activity, including anacardic acid-containing fractions (9, 12, 15).

Preliminary clinical observations in sinonasal disease have suggested that fractionated lentisk oil may represent a promising adjunctive strategy for preventing recurrence of biofilm-associated inflammatory conditions (15). Based on these mechanistic premises, evaluating winterized lentisk oil in patients with vasomotor rhinitis using both symptom-based outcomes (such as SNOT-22) and objective nasal cytological parameters may provide novel insights into the interplay between neurogenic dysfunction, mucosal inflammation, and microbial modulation.

## Materials and methods

### Study design

This study has a prospective, controlled design and aims to evaluate, as its primary endpoint, the effectiveness of the medical device Bactorinol® nasal spray (PharmExtracta S.p.A., Pontenure, Italy) on Peak Nasal Inspiratory Flow (PNIF). Peak Nasal Inspiratory Flow (PNIF) was assessed using a calibrated mechanical peak nasal inspiratory flow meter (GM Instruments), a validated and widely used tool for the objective evaluation of nasal airway patency (16–20). Measurements were performed with the patient seated at rest, using bilateral forced nasal inspiration through a standardized face mask. Three consecutive measurements were obtained, and the highest value was used for analysis. The additional endpoint evaluating the impact of symptoms on daily life was assessed using the SNOT-22 (Sino-Nasal Outcome Test—22 items) questionnaire (21). The secondary endpoints included cytological and functional parameters related to nasal mucosal health. Nasal cytology sampling was performed bilaterally at the inferior aspect of the inferior turbinate using a Rhino-Probe under direct visualization with frontal light and Killian nasal speculum. The collected material was immediately transferred onto glass slides, with one slide prepared for each nostril, air-dried, and subsequently stained using May-Grünwald-Giemsa according to standardized international nasal cytology methodology (22).

Lentisk oil (LO) was supplied by Mediflora® (Pula, Cagliari, Italy). The oil was produced from ripe Pistacia lentiscus berries harvested during winter in southern Sardinia (specific weight: 890 kg/m^3^). As reported by the manufacturer, the drupes underwent mild air dehydration for 20 days before cold pressing, following the procedure described by Mezni et al. (23). The resulting oil was filtered and kept in stainless steel containers at 20 °C until bottling. For the present analyses, the sample was centrifuged at 12,000 rpm for 15 min. The supernatant was then winterized according to Nergiz and Celikkale (24) to remove fatty material and other impurities and improve sample stability (9, 25. Bactorinol® contains lentisk oil (*Pistacia lentiscus* L., berry oil), vitamin E (DL-alpha-tocopherol) as antioxidant, shea butter as vegetable fat, and medium-chain triglycerides.

The evaluation for an allergic etiology, performed before classification as vasomotor rhinitis (VMR), included skin prick testing and measurement of serum total and allergen-specific IgE. Negative skin prick tests and serum allergen-specific IgE results provided no evidence of systemic sensitization to the allergens tested, supporting the nonallergic classification (26, 27).

Cytological findings compatible with biofilm-like structures were identified in samples obtained from the inferior turbinate mucosa and defined as cyan-colored amorphous areas containing morphologically recognizable bacterial aggregates following May-Grünwald-Giemsa staining, according to standardized cytological criteria (22). Additionally, the presence of the supranuclear stria, was examined. Cytological preparations were further analysed for the presence of bacterial elements. The presence of bacteria in the cytology sample can be determined by identifying the various bacterial forms; however, it is not possible to perform a microbiological analysis to identify the specific type of bacteria. Changes in cellularity, including neutrophils, eosinophils, and mast cells, were also recorded. All cytological evaluations were performed by a trained responsible investigator with formal expertise in nasal cytology acquired through dedicated academic training and longstanding clinical experience, including membership in the Italian Academy of Nasal Cytology. Cytological assessments were performed in a non-blinded manner. Quantification was carried out by the responsible physician using a 0–4 Likert-scale, where 0 = absence and 4 = marked abundance. This semiquantitative grading approach is consistent with internationally standardized nasal cytology methodology (22). Individuals with immune disorders, neoplastic diseases, or neurological and/or psychiatric conditions, were considered ineligible for the study. Concomitant topical application of other medications to the nasal mucosa was not permitted during the study period. Eligible participants were consecutively recruited and assigned to treatment or control groups in a 1:1 ratio through block randomization (block size = 4). The randomization sequence was generated using Microsoft Excel by the statisticians involved in the study and was subsequently provided directly to the physicians responsible for participant allocation. Patients in the treatment group received Bactorinol® nasal spray at a dosage of 3 puffs per nostril, 3 times daily for 20 days, while participants allocated to the control group received isotonic saline nasal spray alone, administered according to the same dosing schedule as the investigational medical device, for the same duration. The study was conducted with the approval of the Ethics Committee of the University of Urbino (meeting no. 95, June 26, 2025; study/protocol ID: 116/22.11.2024/BACTORINOL). The study was registered at ClinicalTrials.gov under the identifier NCT07266688. Participant recruitment started on December 1, 2025, and the study was completed on April 20, 2026. All participants provided written informed consent before enrollment.

### Statistical analysis

Independent samples t-tests were conducted to identify potential baseline differences between groups (treated vs. control) for quantitative variables (age, SNOT-22_T0_, PNIF_T0_). Effect sizes were calculated using Cohen's d (28). To ensure the correct application of the Chi-square test, Yates' correction (*χ*^2^Y) was applied where appropriate (29). Outliers were identified using the Tukey method, which detects extreme values by defining lower and upper bounds based on the interquartile range (IQR). Specifically, observations falling below Q1−1.5×IQR or above Q3+1.5×IQR were classified as outlier (30). This approach was adopted given the low number of outliers, which were not attributable to measurement errors (treated group: 1 outlier for SNOT-22 at T_0_, 3 outliers for PNIF at T_0_, and 1 outlier for SNOT-22 at T_20_; control group: 3 outliers for SNOT-22 at T_0_ and 3 outliers for PNIF at T_0_). To reduce the influence of extreme values, a winsorization procedure was subsequently applied, whereby outliers were replaced with the corresponding lower or upper Tukey boundary values (31).

To evaluate the effect of the medical device on nasal inspiratory flow (PNIF) improvement and on the reduction of the impact of vasomotor rhinitis on quality of life, as assessed by the SNOT-22 questionnaire, a repeated-measures General Linear Model (GLM) was performed. Time (pre–post treatment) was included as the within-subject factor, while group and sex was considered as the between-subject factor. Age entered as covariates in the model. In addition, the interaction terms time × group, time × age, and time × sex were included to investigate potential differential effects over time according to group assignment and demographic variables. Pillai's Trace (V) was used as the multivariate test statistic. All tests were performed using Excel (Microsoft 365 MSO, 2512 Build version) and Rstudio (version 4.4.2).

## Results

### Participants

Of the 120 participants initially enrolled, 10 (of whom 8 belonged to the control group and 2 to the treatment group) voluntarily withdrew from the study for personal reasons. Consequently, data from a total of 110 subjects were analyzed, of whom 52.73% (*n* = 58) belonged to the treatment group and 47.27% (*n* = 52) to the control group. At baseline, as showed in Table 1, the two groups were comparable in terms of age (mean age T_0_ = 45.5 ± 14.45 years; mean age C_0_ = 47.1 ± 12.86; *p* = 0.547; Cohen's d = 0.117) and Peak Nasal Inspiratory Flow (PNIF T_0_ = 69.79 ± 22.31 L/min; PNIF C_0_ = 68.85 ± 24.74 L/min; *p* = 0.835; Cohen's d = 0.04).

**Table 1:** Baseline and post-treatment characteristics in the two groups.

Variable	T (*n* = 58)	C (*n* = 52)	*p* (Cohen's d)
Age	45.5 ± 14.45	47.1 ± 12.86	0.547 (0.117)
SNOT - T_0_	41.78 ± 11.77	34.72 ± 13.71	*0.005* (0.55)
PNIF T_0_	69.79 ± 22.31	68.85 ± 24.74	0.835 (0.04)
SNOT - T_20_	29.05 ± 12.48	31.37 ± 14.38	0.373 (0.171)
PNIF T_20_	100.86 ± 27.31	83.17 ± 25.06	*0.001* (*0.677)*

Italicized *p*-values indicate statistical significance (*p* < 0.05).

In contrast, the two groups were not homogeneous for SNOT-22 scores (T_0_ = 41.78 ± 11.77; C_0_ = 34.72 ± 13.71; *p* = 0.005; Cohen's d = 0.55) or sex distribution between groups (females - T_0_= 42.3%; females - C_0_ = 62.1%; *χ*^2^ = 3.539; *p* = 0.036). These findings suggest a higher proportion of females in the control group and greater baseline impairment in quality of life among participants in the treatment group. Participant flow through the study is summarized in the CONSORT diagram (Figure 1).

**Figure 1:**
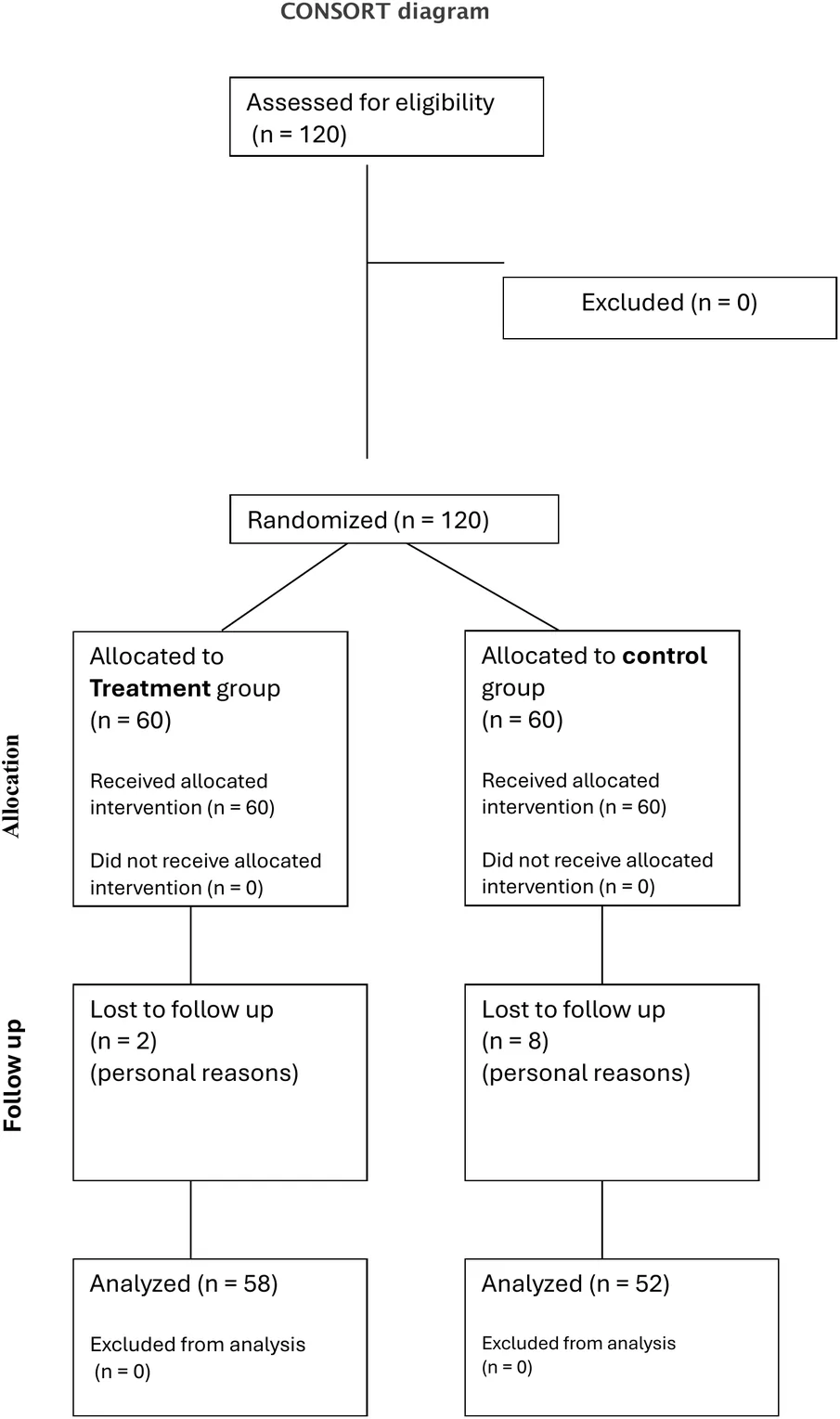
CONSORT flow diagram.

Baseline eosinophil (*χ*^2^ = 112.85; *p* < 0.001), mast cell (*χ*^2^ = 112.39; *p* < 0.001), and neutrophil (*χ*^2^ = 121.02; *p* < 0.001) profiles differed significantly between the two groups, suggesting distinct immunological patterns, consistent with the poorer baseline clinical condition observed in the treatment group in terms of SNOT-22 total score. In the generalized linear model, the interaction term time×group was statistically significant only for neutrophils (*p* = 0.001) and mast cells (*p* = 0.021), indicating that the temporal changes in these outcomes differed significantly between the treated and control groups. The complete profile is shown in Figure 2. The supranuclear stria was absent in 98.1% of cases in the control group and in 89.65% of cases in the treatment group (*χ*^2^Y = 2.00; *p* = 0.104). Baseline cytological findings compatible with biofilm-like structures were detected in 30.77% (*n* = 16) of subjects in the control group and in 50.0% (*n* = 29) of those in the treatment group, showing a significantly higher prevalence in the treatment group before the intervention (*χ*^2^Y = 3.437; *p* = 0.039).

**Figure 2:**
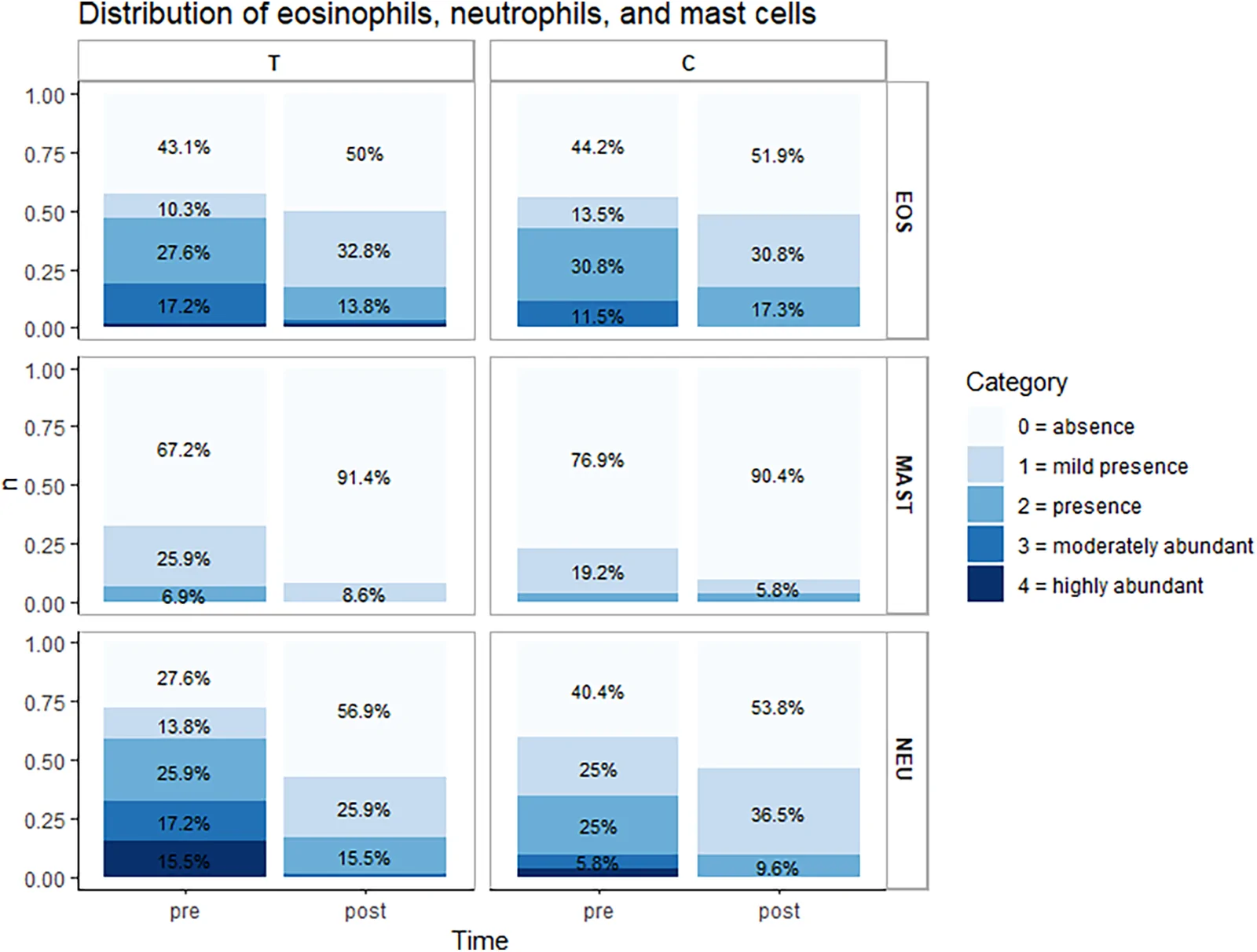
Distribution of eosinophils, neutrophils and mast cells stratified by group (T = treated; C = control) and time (pre; post).

### Treatment effect

In the repeated-measures GLM, a significant effect of time (Pillai's Trace = 0.148; *p* = 0.038) and a significant time × group interaction (Pillai's Trace = 0.306; *p* < 0.001) were observed, whereas the time × age and time × sex interactions were not statistically significant (*p* > 0.05).

Univariate analyses showed that SNOT-22 scores were significantly influenced only by the time × group interaction (*p* < 0.001), indicating a greater reduction in the treatment group compared with the control group, in the absence of a significant main effect of time (*p* = 0.416). In percentage, SNOT-22 scores decreased by 30.5% in the treatment group (T_0_ = 41.78 ± 11.77 vs. T_20_ = 29.05 ± 12.48) and by 9.7% in the control group (C_0_ = 34.72 ± 13.71 vs. C_20_ = 31.37 ± 14.38). The mean reduction in SNOT-22 score in the treatment group was 12.73 points, exceeding the commonly reported minimal clinically important difference (MCID) of approximately 9 points for the SNOT-22. In comparison, the control group showed a mean reduction of 3.35 points, which remained below this threshold, suggesting that the statistically significant between-group difference was also associated with a clinically meaningful improvement in patient-reported sinonasal symptoms.

Notably, the post-treatment SNOT-22 score in the treatment group was lower than that observed in the control group (29.05 vs. 31.37), with an estimated between-group difference in change of −9.06 points (95% CI: −12.61 to −5.51; *p* < 0.001), despite the treatment group presenting with higher baseline values. This reduction is illustrated in Figure 3 through box and violin plots.

**Figure 3:**
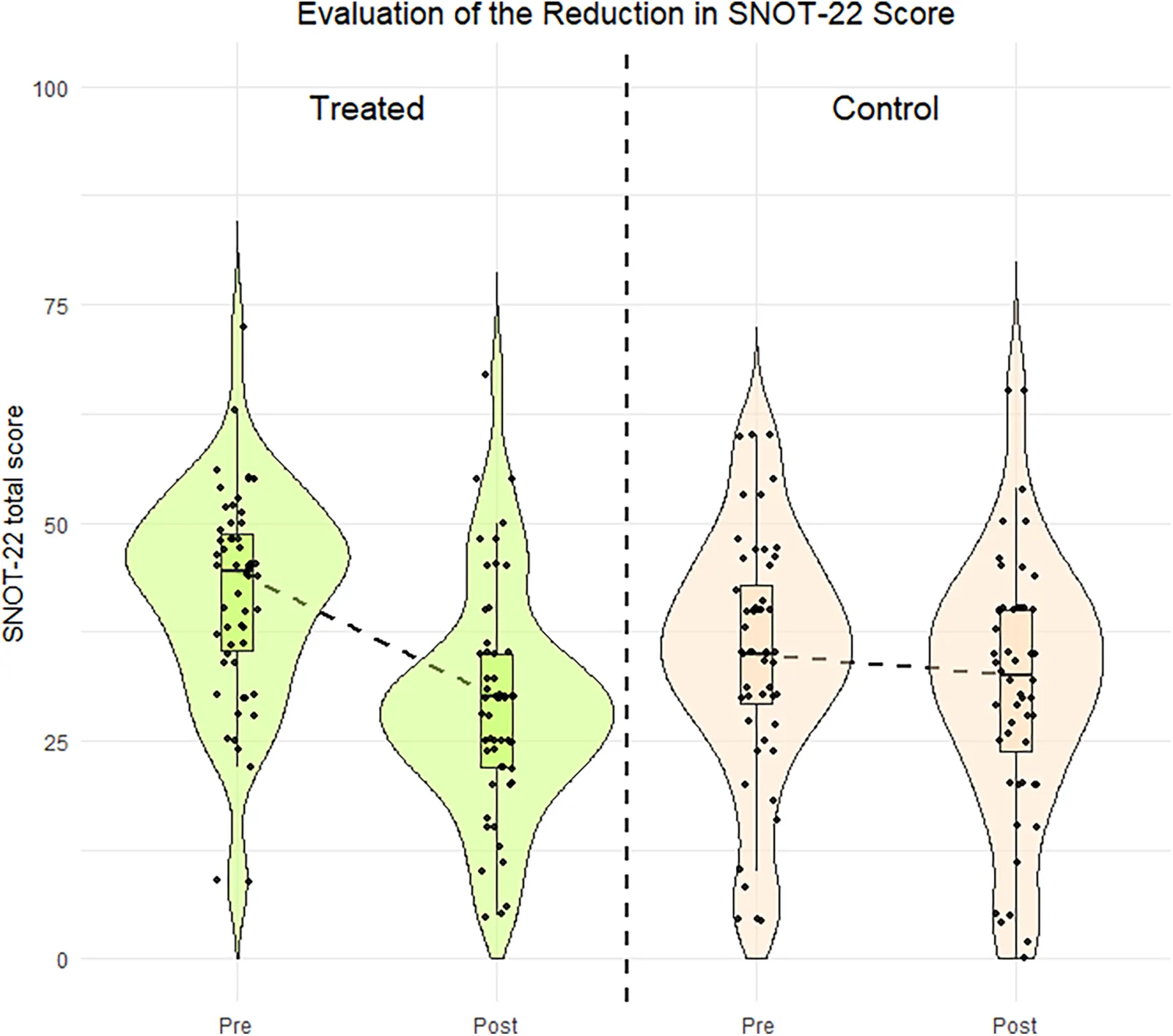
Reduction in SNOT-22 total score between the treated and control groups across the two time points.

For PNIF, a significant main effect of time was observed (*p* = 0.03), indicating an overall improvement over time. However, the significant time × group interaction (*p* = 0.006) suggests a faster and more pronounced increase in the treatment group compared with the control group.

Specifically, PNIF increased by 44.5% in the treatment group (mean PNIF: T_0_ = 69.79 ± 22.31 vs. T_20_ = 100.86 ± 27.31) and by 20.8% in the control group (mean PNIF: C_0_ = 68.85 ± 24.74 vs. C_20_ = 83.17 ± 25.06), with an estimated between-group difference in change of 16.32 L/min (95% CI: 4.78–27.87; *p* = 0.006). Consequently, the post-treatment PNIF value was higher in the treatment group than in the control group, as also shown in Figure 4*.*

**Figure 4:**
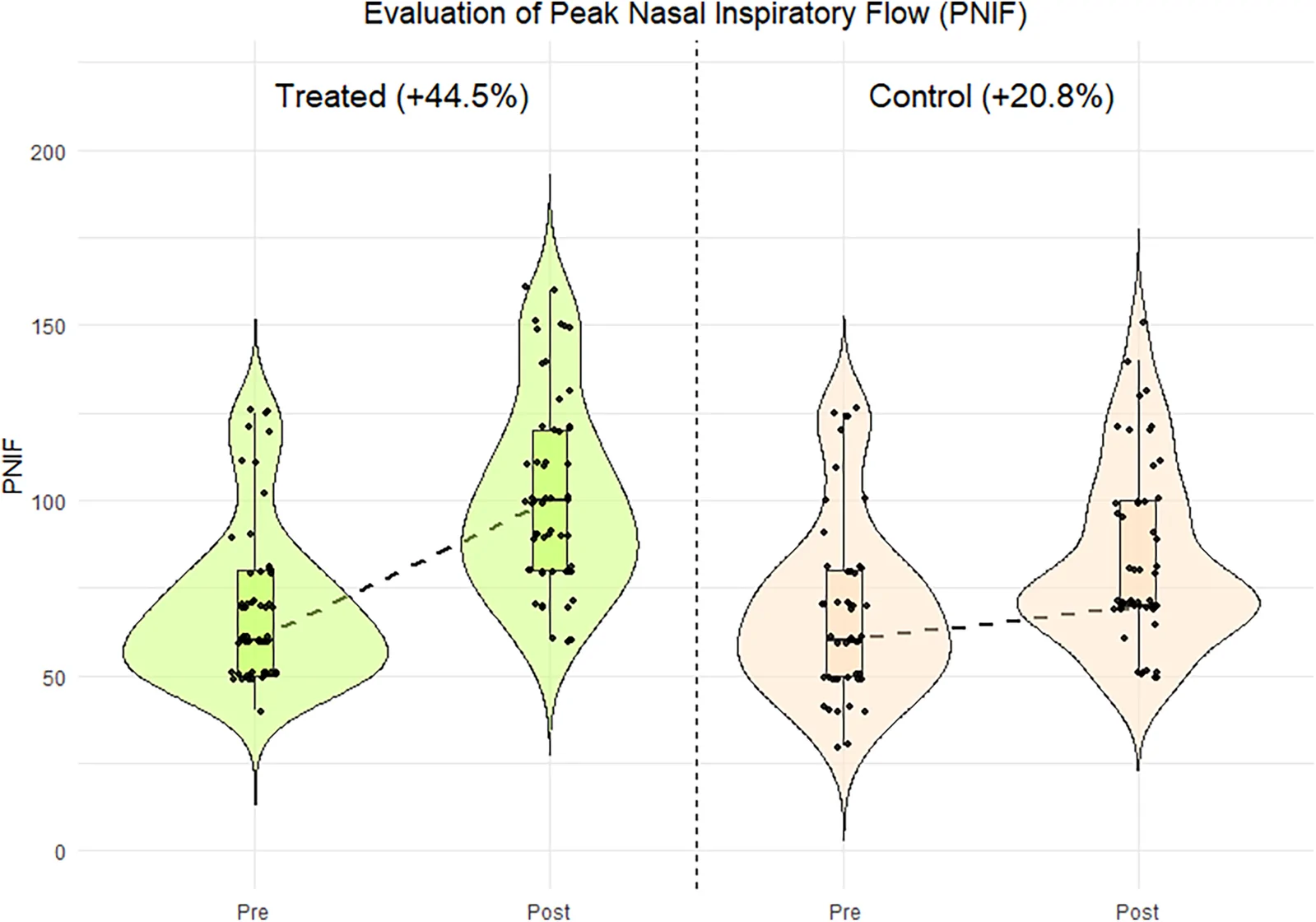
Peak nasal inspiratory flow (PNIF) in both groups, stratified by time (pre- and post-treatment).

Regarding bacterial abundance, it is noteworthy that in the treatment group all subjects with moderate bacterial abundance (15.52%) and high bacterial abundance (6.89%) showed a reduction over time. In fact, at T_20_ the treatment group no longer presented any subjects with bacterial abundance above the mild level. In the control group, although a smaller proportion of subjects initially exhibited moderate (3.85%) and high (1.92%) bacterial abundance, reductions were observed only among those who had high abundance at baseline. Conversely, subjects with moderate abundance remained unchanged (3.85%). In the GLM analysis, the interaction term time × group was statistically significant also on bacterial abundance (*p* = 0.002), highlighting a significant treatment effect. These patterns are illustrated in Figure 5.

**Figure 5:**
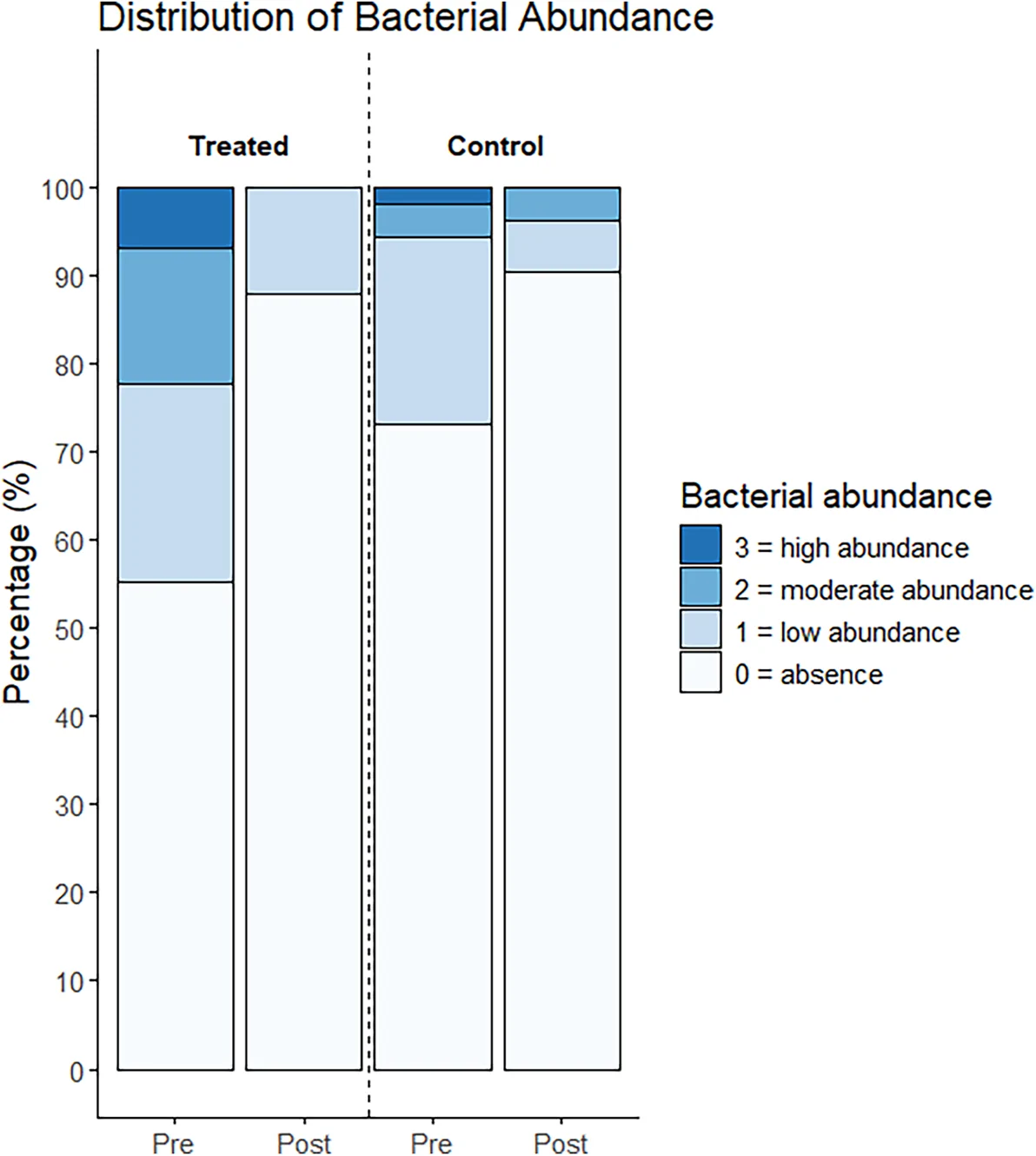
Distribution of bacterial abundance in the two groups over time (pre- and post-treatment).

Although the treated group presented a higher of biofilm-like cytological findings baseline prevalence of of biofilm-like cytological findings compared to the control group (50.0% vs. 30.77%), as shown in Figure 6, a lower prevalence was observed at the end of the study in the treated group. This pattern indicates a greater reduction over time in the treated group, consistent with the significant group-by-time interaction. This pattern indicates a greater reduction over time in the treated group, consistent with the significant group-by-time interaction (*p* = 0.001).

**Figure 6:**
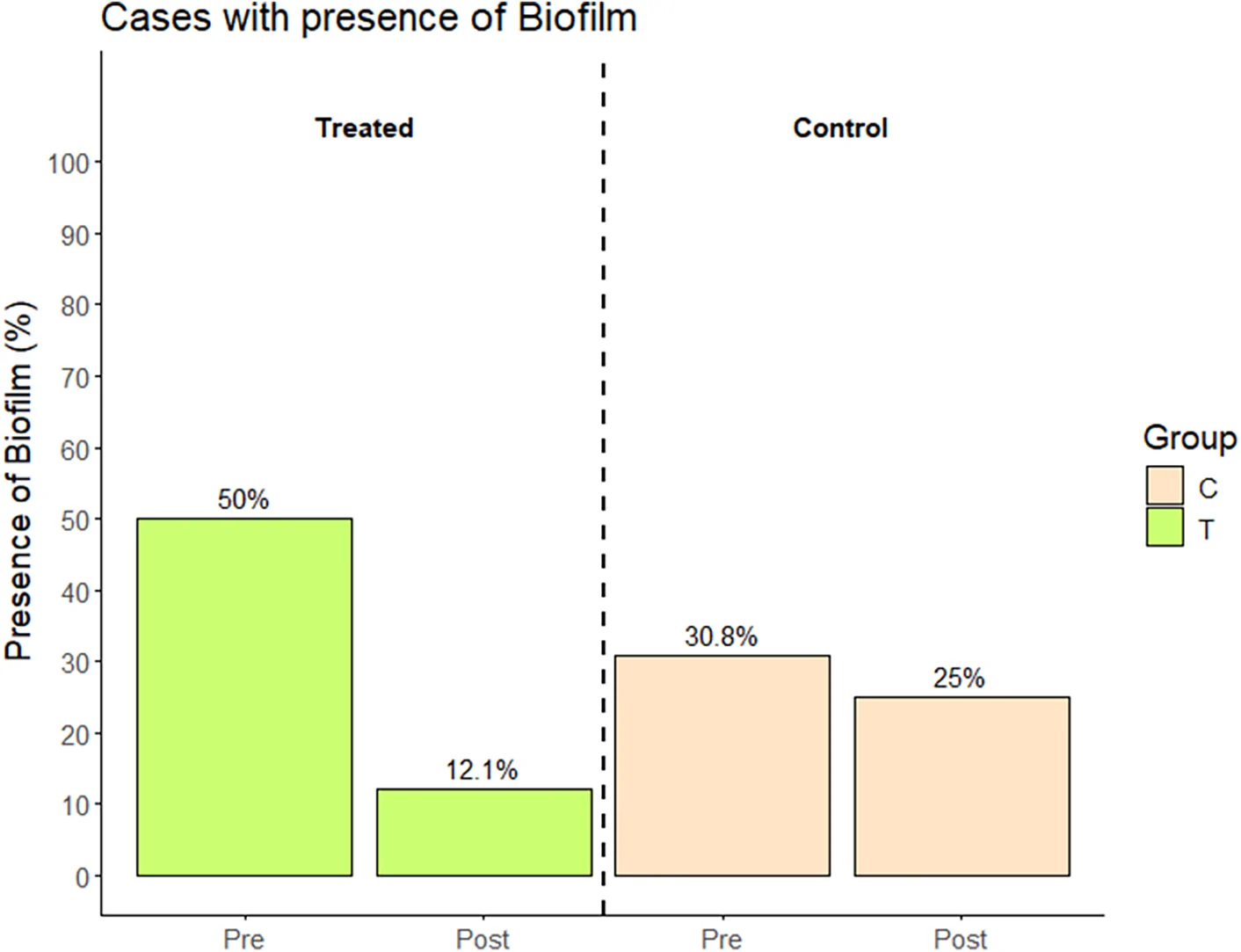
Reduction in the number of cases with cytological findings compatible with biofilm-like structures presence in the two groups.

## Discussion

This prospective controlled study suggests that treatment with winterized *Pistacia lentiscus* oil nasal spray may provide clinically meaningful benefits in patients with vasomotor rhinitis (VMR), with improvements observed across both subjective and objective parameters. Compared with controls, treated patients showed a greater reduction in symptom burden, as measured by SNOT-22, together with a more pronounced increase in Peak Nasal Inspiratory Flow (PNIF), indicating improved nasal patency and functional airflow.

VMR remains a diagnostically challenging and pathophysiologically heterogeneous condition. Traditionally, its underlying mechanisms have been attributed primarily to autonomic dysregulation and neurogenic hyperreactivity, resulting in abnormal vasodilation, glandular hypersecretion, and exaggerated responses to non-specific triggers (1) (2), (3), (4),. However, this classical neurovascular interpretation may not fully explain the variability in clinical presentation, chronicity, and treatment responsiveness observed in routine practice. The present findings raise the possibility that, at least in selected patients, local mucosal microbial burden and biofilm-related phenomena may contribute to symptom persistence or amplification.

The role of bacterial biofilms has been extensively characterized in chronic rhinosinusitis, where microbial communities embedded in extracellular polymeric matrices are known to promote persistence, chronic inflammation, and reduced responsiveness to antimicrobial therapies (7, 8). While equivalent evidence in VMR remains limited, the significant reduction in cytologically detected bacterial abundance and prevalence of biofilm-like cytological findings observed in the treated group suggests that microbial factors may represent an underexplored component in some phenotypes currently classified under the broad umbrella of non-allergic rhinopathy. This hypothesis should be interpreted cautiously, as cytology provides indirect morphological evidence rather than microbiological identification, yet the observed trends remain biologically interesting.

An additional relevant observation concerns inflammatory cellularity. The significant treatment-associated changes in neutrophils and mast cells may suggest that the intervention influences not only microbial burden but also local inflammatory signaling. Mast cells are increasingly recognized as relevant effectors not exclusively in allergic disease, but also in neurogenic and non-IgE-mediated mucosal hyperreactivity, where interactions between neural pathways, epithelial barrier integrity, and innate immune activation may contribute to symptom generation (3, 4),. The reduction of these cellular markers alongside symptomatic improvement supports the possibility of a broader mucosal regulatory effect.

A plausible mechanistic explanation may lie in the biological properties of winterized *Pistacia lentiscus* oil. Experimental evidence has shown that lentisk oil exerts antimicrobial activity against upper airway pathogens, including *Streptococcus* species and *Staphylococcus aureus*, with effects attributed to unsaturated fatty acids and bioactive anacardic acid derivatives (9–13). Winterization enhances the concentration of lipophilic fractions potentially involved in anti-biofilm activity, improving the theoretical ability of the preparation to interfere with microbial adhesion and persistence (9, 15). The present clinical findings are coherent with this mechanistic framework.

Beyond direct antimicrobial effects, an emerging conceptual framework may further support interpretation of these findings. Increasing evidence suggests that nutraceutical and plant-derived bioactive compounds may act as ecological modulators of host-associated microbial ecosystems rather than functioning solely as conventional antimicrobial agents. This concept has already been explored in gastrointestinal settings, where selected nutraceutical interventions may exert prebiotic-like or microbiota-modulating effects, influencing host inflammatory responses and microbial ecological balance (32). Extending this perspective to the upper airway mucosa may open new translational avenues, particularly for chronic inflammatory conditions where dysregulated host–microbe interactions could contribute to symptom persistence.

Some methodological limitations should be acknowledged. First, baseline heterogeneity between groups was present, particularly for SNOT-22 scores, sex distribution, inflammatory cytology, and prevalence of biofilm-like cytological findings, suggesting that the treatment group initially presented a more compromised clinical profile. Although statistical adjustment was applied, residual confounding cannot be excluded. Second, cytological assessment identifies morphological bacterial presence and biofilm-like structures but does not allow microbiological characterization or direct microbiome profiling. Therefore, mechanistic conclusions regarding microbial composition remain speculative. Third, cytological assessments were performed in a non-blinded manner. Since semiquantitative cytological grading relies on visual interpretation, observer bias cannot be completely excluded, despite the use of a single experienced investigator and standardized sampling, staining, and scoring procedures. Fourth, the follow-up period was relatively short, limiting conclusions regarding the durability of the response and recurrence prevention. Fifth, although the findings are encouraging, the study design does not allow definitive causal attribution between microbial reduction and clinical improvement. Sixth, the intervention and control preparations were not fully placebo-matched formulations. Although both sprays were administered according to the same dosing schedule, potential differences in sensory or physical characteristics, including appearance, odor, viscosity, or administration experience, may have contributed to partial unblinding and expectation bias. Future randomized controlled trials should consider the use of a fully matched placebo formulation to minimize this potential source of bias. Finally, the study was conducted in a relatively selected clinical population, which may limit the generalizability of the findings to the broader and heterogeneous population of patients with non-allergic rhinitis. Larger multicenter studies including more diverse patient populations are warranted.

Nevertheless, the strengths of this study include the integration of subjective symptom assessment, objective airflow measurement, and cytological evaluation, offering a multidimensional view of treatment response. This approach may be particularly valuable in disorders such as VMR, where purely symptom-based evaluation risks oversimplifying heterogeneous pathophysiological processes.

## Conclusion

Winterized *Pistacia lentiscus* oil nasal spray was associated with significant improvements in symptoms, nasal airflow, inflammatory cytology, bacterial abundance and cytological findings compatible with biofilm-like structures in patients with vasomotor rhinitis. While VMR has traditionally been interpreted primarily as a neurovascular disorder, these findings suggest that microbial and mucosal inflammatory factors may contribute to disease expression in selected patients. The antimicrobial and anti-biofilm properties of winterized lentisk oil provide a biologically plausible explanation for the observed effects.

These results should be considered exploratory but support the concept that phytotherapeutic interventions with microbial-modulating properties may represent a promising complementary strategy in the management of chronic non-allergic nasal disorders. Future randomized studies incorporating microbiological culture, molecular microbiome profiling, and longer-term follow-up are warranted to better define mechanisms, identify responder phenotypes, and confirm clinical applicability.

## Data Availability

The data underlying the results presented in this study are available from the corresponding author upon reasonable request, in accordance with applicable ethical and privacy restrictions.
